# A study on the factors influencing the utilization of public health services by China's migrant population based on the Shapley value method

**DOI:** 10.1186/s12889-023-17193-3

**Published:** 2023-11-24

**Authors:** Zhonghua Suo, Lina Shao, Ying Lang

**Affiliations:** Department of Health Policy and Management, School of Humanities and Management, Ningxia Medical University, No. 1160, Shengli Street, Xingqing District, Yinchuan, Ningxia China

**Keywords:** Migrant population, Public health service utilization, Shapley value method, Anderson model, Influencing factors

## Abstract

**Background:**

The health of migrants has received significant global attention, and it is a particularly significant concern in China, which has the largest migrant population in the world. Analyzing data on samples from the Chinese population holds practical significance. For instance, one can delve into an in-depth analysis of the factors impacting (1) the health records of residents in distinct regions and (2) the current state of family doctor contracts. This study explores the barriers to access these two health services and the variations in the effects and contribution magnitudes.

**Methods:**

This study involved data from 138,755 individuals, extracted from the 2018 National Migration Population Health and Family Planning Dynamic Monitoring Survey database. The theoretical framework employed was the Anderson health service model. To investigate the features and determinants of basic public health service utilization among the migrant population across different regions of China, including the influence of enabling resources and demand factors, *x*^2^ tests and binary logistic regression analyses were conducted. The Shapley value method was employed to assess the extent of influence of each factor.

**Results:**

The utilization of various service types varied among the migrant population, with significant regional disparities. The results of the decomposition of the Shapley value method highlighted variations in the mechanism underlying the influence of propensity characteristics, enabling resources, and demand factors between the two health service types. Propensity characteristics and demand factors were found to be the primary dimensions with the highest explanatory power; among them, health education for chronic disease prevention and treatment was the most influential factor.

**Conclusion:**

To better meet the health needs of the migrant population, regional barriers need to be broken down, and the relevance and effectiveness of publicity and education need to be improved. Additionally, by considering the education level, demographic characteristics, and mobility characteristics of the migrant population, along with the relevant health policies, the migrant population needs to be guided to maintain the health records of residents. They should also be encouraged to sign a contract with a family doctor in a more effective manner to promote the equalization of basic health services for the migrant population.

## Background

In July 2022, the World Refugee and Migrant Health Report of the World Health Organization [1] showed that millions of refugees and migrants under vulnerable conditions, such as low-skilled migrant workers, have poorer health compared to host populations. Migration and displacement are significant factors that affect the health of individuals and hinder the achievement of health-related sustainable development goals. Therefore, addressing the health needs of migrants requires global attention. Some studies [2, 3] have classified migration into international and internal migration. Internal migration refers to the movement of people within their country of origin to another region, province, or city. It occurs commonly in countries and regions across the world, including the United States, Europe, and China [4–6]. Some studies have shown that migrants often face higher health risks compared to residents [7]. Migrants also lose social capital in their hukou location and encounter discrimination in employment opportunities and social welfare entitlements at their site of migration [8]. Additionally, they are frequently excluded from the local health system and cannot fully utilize the available health service resources. To tackle these concerns, a study has identified that numerous developed countries and regions have enacted regulations pertaining to public health services and have established management service packages concerning the employment and living conditions of migrants [9]. For instance, the European Union takes into account the needs of migrants when crafting policies and executing programs, with a focus on managing infectious diseases, maternal and child health, occupational health, violence, and migrant health indicators [10]. In a similar vein, India has enacted the Migrant Workers and Interstate Migrant Workers Act [11]. Several studies have demonstrated that institutional, national, and local public health measures play a pivotal role in safeguarding the right to health for migrant populations [12].

China has the largest migrant population in the world, which includes 376 million people, according to the seventh population census data of China [13]. The influx of migrants has led to the integration of resources in these areas and contributed to regional economic development. However, migrants are also exposed to greater health risks. Thus, to protect the right to health of migrants, China has implemented several regulations and initiatives. In 2014, the former National Health and Family Planning Commission, along with other relevant departments, issued the Guiding Opinions on Basic Public Health Services and Family Planning Management for the migrant population. These guidelines prioritized six basic public health services for migrant populations, including the vaccination of children, prevention and control of infectious diseases, maternal and child healthcare, health records, family planning, and health education [2]. Additionally, the outline of the "Healthy China 2030" Plan [14] emphasizes the importance of providing equal access to basic health services for the migrant population. However, the level of economic development, migrant population management policies, and health insurance payment methods vary across different regions of China [15]. These variations can hinder equal access to basic health services for the migrant population. The access of migrants to social benefits, such as health insurance, is closely related to the unique household registration system [16]. While most migrants have social health insurance, there is a conflict between using health services across regions and the localized and fragmented management of health insurance [16, 17]. This conflict decreases flexibility and increases difficulties in utilizing social health insurance in their localities, leading to significant spatial and regional disparities. However, it is not known whether the current basic public health services provided by China can satisfy the needs of the migrant population. The hindrances and limitations that prevent the migrant population from accessing basic public health services in areas with an influx of migrants need to be identified. These issues and concerns can be determined by studying the utilization of public health services by the migrant population in different regions of China.

Basic public health services include the establishment of the health records of residents and family doctor contracts, among others. The health records of residents serve as standardized records that document the medical and health services provided to them [18]. Family doctors can offer health counseling, basic medical care, and other essential public health services to contracted residents [19]. Therefore, it is crucial to assess the current status of health records and family doctor contracting services for the migrant population and analyze the factors influencing them. The data can provide information on whether the public health service needs of migrants are met [20]. These two types of services strongly influence the implementation of other items [21].

However, previous studies [22–26] onthat investigated the health records of migrant populations and their access to family doctors had some limitations. These studies did not include all influencing factors, such as the reason for migration, duration of migration, and variables reflecting the health needs of the migrant population, such as their health status, health education [20]. Additionally, the studies failed to consider the different types of health insurance and categorized it broadly as the presence or absence of health insurance. This limitation hindered the validation of the effect of different types of health insurance on the utilization of basic public health services by migrant populations [23]. Some studies did not analyze any influencing factors, while others only provided qualitative analysis. Some studies conducted quantitative analysis, but lacked a theoretical analysis framework, resulting in a lack of systematic and comprehensive analysis. Furthermore, most researchers investigated China's migrant population in a specific province or region only [27–29]. They did not examine the regional differences in the health of the migrant population at a macro level. Some studies highlighted the presence of regional or transnational differences among migrant groups [30, 31]. Considering that China's migrant population is widely distributed, their health status is significantly influenced by the economy and medical services of a region. Many studies only focused on specific subgroups, such as elderly migrants [26], ethnic minorities [32], and rural inflows [33], but ignored the overall migrant population. Previous studies primarily used methods such as statistical description, the *x*^*2*^ test, and logistic regression to examine the current state of health service utilization among the migrant population [21–26]. However, these studies did not quantitatively assess the degree of contribution or influence of each factor that led to variations in basic public health services among migrant populations across different regions. In this study, we addressed this research gap. Some studies identified health inequalities between the migrant population and the non-migrant population [34]. The rate of acceptance of health education and participation in insurance schemes is lesser among the migrant population compared to the local population [35]. Thus, it is important to find effective strategies to study the differences in basic public health services among migrant population groups. Since different regions attract different migrant populations with varying quality compositions [36, 37], addressing the differences in health service utilization among migrant populations in different regions is important. In this study, we assessed the factors that contribute to inter-regional health disparities among migrant populations in China, which is important for the well-being of the migrant population. Anderson's medical and health service model is the best theoretical model for studying health service utilization behavior [38]. In this research, we applied the Andersen behavioral model framework from 2013 to categorize and scrutinize the utilization of basic public health services and the factors influencing it among the migrant population, considering a systematic perspective. Furthermore, we employed the Shapley value decomposition method, which draws upon the concept of cooperative games, to gauge the contribution of each explanatory variable to the disparities in the variables under examination. At present, numerous scholars have been using the Shapley value method to explore diverse aspects, such as disparities in health outcomes between urban and rural residents, the selection of residential institutions, higher education, and health, along with the factors influencing these phenomena [39, 40].

In this study, the 2018 National Migrant Population Health and Family Planning Dynamic Monitoring Survey Database was analyzed using Anderson's health service utilization model. We assessed whether there are differences in the utilization of basic public health services among migrant populations in the eastern, central, western, and northeastern regions of China. Additionally, we identified the shortcomings in the supply of basic health services for migrant populations and the obstacles faced in providing these services to the migrant population in different regions. We investigated the factors influencing the utilization of basic public health services for the migrant population in China. We also determined the degree of contribution of each influencing factor, focusing on the three dimensions of the migrant population's tendency characteristics, enabling resources, and demand factors. By identifying the main factors that contribute to the population health records and family doctor contracts of the migrant population, we provided valuable information, which can be used to decide suitable interventions and optimize the allocation of medical resources. We also investigated whether the differences in the types of health insurance offered to the migrant population, based on geographic location, result in variations in the quality of health services provided. Based on the findings, we proposed targeted recommendations to address any disparities. We evaluated the health status of the migrant population across diverse geographical regions. Our discoveries could serve as a benchmark for adjusting the distribution of healthcare resources and identifying priority areas for enhancing the well-being of migrants. These findings may contribute to improving the accessibility and equity of healthcare services for migrants. Furthermore, policymakers and healthcare providers can utilize this information to formulate tailored healthcare policies that address the specific needs of migrants. This study can aid other countries in emphasizing critical influencing factors, mitigating health disparities, enhancing the effective management of the impact of the migrant population on public health, comprehending the characteristics and requirements of migrants, and creating and implementing targeted public health policies. Additionally, understanding the weights and differences of different influencing factors can help other countries optimize resource allocation when resources are limited and meet the needs of migrants for public health services.

## Data and methods

### Data sources

We used the latest data from the China Migrants Dynamic Survey (CMDS) conducted in 2018. The survey targeted individuals who resided in the local area for at least one month, were not members of the district's household, and were 15 years old or older. The questionnaire covered various aspects of the migrant population and household members, including basic demographic information, migration patterns, information on employment and social security, income and expenditure, residence, basic public health services, and other relevant factors. The survey included 152,000 individuals. From the database, 138,755 migrants were selected as research participants, ensuring no missing research variables. The CMDS 2018 used a stratified, multi-stage, PPS sampling method proportional to the size for sampling. Provinces, cities, and districts were categorized into four regions (east, central, west, and northeast) based on the division criteria set by the State Council, as shown in Table 1.

**Table 1:** The stratified variation scale of the CMDS areas in 2018

District	Province/city/region
Eastern Region	Beijing, Hebei, Tianjin, Shandong, Jiangsu, Zhejiang, Shanghai, Fujian, Hainan, Guangdong
Western Region	Inner Mongolia Autonomous Region, Xinjiang Production and Construction Corps, Xinjiang Uygur Autonomous Region, Shaanxi Province, Ningxia Hui Autonomous Region, Gansu Province, Chongqing Municipality, Qinghai Province, Tibet Autonomous Region, Sichuan Province, Guizhou Province, Guangxi Zhuang Autonomous Region, Yunnan Province
Central Region	Shanxi, Hubei, Henan, Anhui, Jiangxi, Hunan
Northeast	Heilongjiang, Liaoning and Jilin provinces

### Research variables

The independent variables included the following: (1) the characteristics of the migrant population, including demographic factors (gender, age, etc.) and social structure factors (education level, marital status, household registration, and migration characteristics); (2) enabling resources (type of health insurance, place of coverage, and income level); (3) demand factors (self-rated health status, health education for chronic disease prevention and treatment, and two-week prevalence). By considering the data on the establishment of health records of migrants and the contract of family doctors as dependent variables, those who answered "yes, already established" were regarded as established and assigned a value of 1; those who answered "no construction, never heard of it" and "no construction, but heard of it" were regarded as not established and assigned a value of 0. These values and data were used to construct the basic public health service utilization model of the migrant population.

### Research methods

SPSS 25.0, Stata 14.0, and Excel 2019 were used for data screening, cleaning, statistical analysis, and data tabulation. The Chi-square test and binary logistic regression model were used to conduct inter-group comparisons, and propensity factors, enabling resources, and demand factors were introduced one at a time to elucidate the effects of different factors on regional differences in health service utilization among the Chinese migrant population. All results were considered to be statistically significant at *P* < 0.05. The regression equation was fitted as follows:$y={\beta }_{0}+{\beta }_{1x1}+{\beta }_{2x2}+{\beta }_{3x3}+{\beta }_{4x4}+\varepsilon$ where y indicates the resident health records of the migrant population, × 1 indicates the characteristics of the migrant population tendency (such as gender), × 2 and × 3 indicate enabling resources (medical insurance type, income, etc.), and × 4 indicates the demand factors, such as self-rated health status. The Shapley value decomposition method based on regression analysis was used to quantify the degree of contribution of each dimension and its variables [40], i.e., the weight of each influencing factor. It can not only explain the individual contribution rate of influencing factors to the difference of dependent variables but also combine and decompose the overall contribution of a certain category of influencing factors. We identified the dimensions of the migrant population's tendency characteristics, enabling resources, and demand factors that had the greatest weight on the interpretation of the establishment of the health records of residents, i.e., we determined the degree of impact. After performing the regression analysis, we included the statistically significant factors of binary logistic regression and ran the following commands on the Stata software: shapley2, stat (r2) group (X1, X2 X3, X4 X5 X6). After running the command, we obtained the Shapley value and the corresponding percentage (contribution rate). The same method was used for analyzing the data on family doctors.

## Results

### Sample characteristics

According to the survey respondents, the migrant population in the eastern region accounted for 47.06% (65,295 cases), while that in the central, western, and northeastern regions accounted for 16.03% (22,247 cases), 29.97% (41,580 cases), and 6.94% (9,633 cases), respectively. Regarding propensity characteristics, most migrants in each region were 25–54 years old. The gender distribution showed a higher proportion of males. Employment status was dominated by employees and self-employed laborers. The highest proportion of the migrant population with a bachelor's degree or above was found in the eastern region, while the western region had the largest number of agricultural households. Most migrants were married across all regions. The scope of mobility differed among regions; the highest proportion of inter-provincial mobility was observed in the eastern region (73.47%). The proportions of intra-provincial cross-city (43.13%), intra-city cross-county (35.53%), and cross-province (21.34%) mobility were ordered in the central region. The western region and the northeastern region had a similar proportion of scope of mobility. Regarding resource characteristics, the migrants in the four regions were mainly a part of the new rural cooperative medical insurance and urban workers' basic medical insurance schemes. The proportion of migrants under the urban workers' basic medical insurance scheme was the highest in the eastern region (33.98%). Most participants in the eastern, central, and western regions were registered in the household, followed by local participants, with the highest proportion in the western region. In the northeastern region, the proportion of local participants (50.92%) was higher than that of registered participants (48.42%). Significant variations existed in the income levels of migrants across the four regions. In the eastern region, half of the migrant population had either high or middle income. Conversely, the central region had a greater concentration of middle-income and middle-to-high-income groups. Meanwhile, the western and northeastern regions had a higher share of low-income and lower-middle-income groups in comparison to the proportion of high-income groups. In terms of demand factors, the highest percentage of individuals who assessed their health as 'health' was observed in the eastern region, reaching 98.90%. In contrast, the acceptance rate of health education for chronic disease prevention and treatment was the lowest in the eastern region (23.75%), which was substantially lower than that recorded in the central and western regions and also lower than the national average (30.60%). The northeastern region had the highest two-week prevalence rate among the migrant population (5.21%), followed by 2,076 reported cases of illness in the western region. Further details are provided in Table 2.

**Table 2:** Basic characteristics of the migrant population in different regions (n, %)

Variable	Classification	East	Central	West	Northeast
Age (years)	15–24	13143(20.13)	2210(9.93)	7085(17.04)	1191(12.36)
	25–34	20784(31.83)	8483(38.13)	12496(30.05)	2415(25.07)
	35–44	17781(27.23)	6359(28.58)	11017(26.5)	2421(25.13)
	45–54	10327(15.82)	4050(18.2)	7733(18.6)	2033(21.1)
	55–59	1372(2.10)	524(2.36)	1276(3.07)	519(5.39)
	≥ 60	1888(2.89)	621(2.79)	1973(4.75)	1054(10.94)
Gender	male	39382(60.31)	11409(51.28)	24120(58.01)	5349(55.53)
	female	25913(39.69)	10838(48.72)	17460(41.99)	4284(44.47)
Employment status	employee	39171(68.97)	9057(47.39)	18072(55.07)	4447(64.26)
	employer	3590(6.32)	1510(7.9)	2229(6.79)	567(8.19)
	Self-employed worker	13676(24.08)	8478(44.36)	11996(36.56)	1852(26.76)
	other	354(0.62)	68(0.36)	518(1.58)	54(0.78)
Education	Primary school or below	13431(20.57)	2816(12.66)	10923(26.27)	2310(23.98)
	Junior high school	22738(34.82)	9479(42.61)	14705(35.37)	4190(43.5)
	High school/technical secondary school	14808(22.68)	5711(25.67)	8640(20.78)	1761(18.28)
	Junior college	7905(12.11)	2767(12.44)	4638(11.15)	791(8.21)
	Bachelor degree or above	6413(9.82)	1474(6.63)	2674(6.43)	581(6.03)
Nature of household registration	Agriculture	46627(71.41)	15475(69.56)	31767(76.4)	6874(71.36)
	non-agricultural	18668(28.59)	6772(30.44)	9813(23.6)	2759(28.64)
Marital status	Married	56194(86.06)	19191(86.26)	35219(84.70)	8078(83.86)
	Not married	9101(13.94)	3056(13.74)	6361(15.3)	1555(16.14)
Migration Range	across provinces	47975(73.47)	4748(21.34)	18584(44.69)	3865(40.12)
	Within the province across the city	11710(17.93)	9595(43.13)	15064(36.23)	3678(38.18)
	intercity	5610(8.59)	7904(35.53)	7932(19.08)	2090(21.7)
Reason for migration	migrant worker/job	47239(72.35)	10080(45.31)	24282(58.40)	5936(61.62)
	Go into business	10364(15.87)	8546(38.41)	9325(22.43)	1232(12.79)
	Accompanying family members	5170(7.92)	2138(9.61)	4482(10.78)	1368(14.20)
	other	2522(3.86)	1483(6.67)	3491(8.40)	1097(11.39)
Duration of migration (years)	0 ~ 4	32613(49.95)	9170(41.22)	19589(47.11)	3671(38.11)
	5 ~ 9	16272(24.92)	7716(34.68)	12231(29.42)	2717(28.21)
	≥ 10	16410(25.13)	5361(24.1)	9760(23.47)	3245(33.69)
Type of medical insurance	urban and rural residents	6523(9.87)	2535(11.63)	6291(15.70)	454(5.32)
	NICM	33788(51.14)	13439(61.65)	23278(58.09)	5195(60.88)
	Urban dweller	3169(4.8)	1865(8.56)	3191(7.96)	1206(14.13)
	Urban workers	22454(33.98)	3933(18.04)	7182(17.92)	1653(19.37)
	Socialized medicine	140(0.21)	27(0.12)	131(0.33)	25(0.29)
Insured places	Local	24256(36.71)	5062(23.22)	5179(15.05)	6062(50.92)
	domicile	41131(62.24)	16392(75.19)	28586(83.05)	5765(48.42)
	elsewhere	695(1.05)	347(1.59)	655(1.90)	79(0.66)
Income level	I	10137(15.52)	5143(23.12)	13548(32.58)	3656(37.95)
	II	9889(15.15)	4052(18.21)	8825(21.22)	2081(21.6)
	III	12355(18.92)	4594(20.65)	7988(19.21)	1821(18.9)
	IV	20133(30.83)	6095(27.4)	8382(20.16)	1629(16.91)
	V	12781(19.57)	2363(10.62)	2837(6.82)	446(4.63)
Self-rated health status	unhealthy	715(1.10)	395(1.78)	1310(3.15)	642(6.66)
	healthy	64580(98.90)	21852(98.22)	41580(96.85)	9633(93.34)
Health education for chronic disease prevention and treatment		15510(23.75)	7933(35.66)	16146(38.83)	2864(29.73)
two-week prevalence		2681(4.11)	502(2.26)	2076(4.99)	502(5.21)

Income levels are classified into five grades based on the average monthly household income. These grades are as follows: Grade I (≤ 4,000 yuan), Grade II (4,001 yuan to 5,500 yuan), Grade III (5,501 yuan to 7,111 yuan), Grade IV (7,112 yuan to 10,000 yuan), and Grade V (> 10,000 yuan). NICM stands for New Rural Cooperative Medical Insurance

### Status of utilization of basic public health services for the migrant population in different regions

The central and western regions had higher rates of establishing health records, at 39.61% and 35.25%, respectively. However, in the total sample, 54.39% of migrants did not establish health records, and 12.43% were unsure about establishing them. A significant difference was found in the establishment rates of health records among migrant populations in different regions (*x*^2^ = 1479.48, *P* < 0.05). The western region had the highest rate of family doctor registration among the migrant population, at 20.59%, while the eastern region had the lowest rate at 17.13%. Significant differences were found in the rates of family doctor registration among the migrant population in different regions ( *x*^2^= 708.68, *P* < 0.05).

### Results of binary logistic regression analysis of basic public health services for the migrant population in different areas

We employed a binary logistic regression model to assess the correlation between the establishment of health records for migrants in distinct regions and several factors. The dependent variable denoted whether the migrant population had established a health record (No = 0, Yes = 1). The independent variables encompassed propensities, enabling resources, and demand-related factors. The findings from the analysis revealed that the likelihood of establishing health records was lower among married individuals, those with agricultural household registration, those in good health, individuals with a high school or secondary education, those aged 60 years or older, those who had received health education regarding chronic disease prevention and treatment, those with various types of health insurance, those with specific employment status, and those who migrated for business or family reasons. The rate of health record establishment for migrant populations exhibited regional and demographic disparities. In the central region, factors linked to higher health record establishment rates included female gender, agricultural household registration, marital status, diverse education levels, good health, ages 55–59, receipt of health education concerning chronic diseases, and having insurance coverage and varying degrees of mobility. In the western region, the factors that increased the chances of establishing a health record included being healthy, locally insured, inter-provincial migrant populations, self-employed laborers, agricultural households, and married migrants. In the northeastern region, a higher rate of establishing health records was associated with being healthy, having a two-week prevalence, receiving health education on chronic disease prevention and control, agricultural households, and being married. These findings are summarized in Table 3. The outcomes of family doctor appointments revealed that in the eastern region, migrants were more inclined to schedule appointments with a family doctor if they were married, held agricultural household registration, were self-employed, possessed new rural cooperative medical insurance, received health education on chronic disease prevention and treatment, and were in good health. Furthermore, the duration of their mobility increased with higher education levels. Moreover, in the central region, the rate of enrolling in health insurance among the migrant population was higher for those who were locally insured, married, and associated with agricultural households. In the western region, factors influencing family doctor contracting services among the migrant population included gender, educational attainment, marital status, age, education on the prevention and treatment of chronic diseases, income level (middle and high), health insurance (urban and rural, urban residents, urban workers, and public medical care), local insurance participation, scope of mobility, and reasons for mobility (all with *P* < 0.05). Among these factors, the rate of appointing a doctor was higher among those migrants who received education on the prevention and treatment of chronic diseases, had a higher income, participated in local insurance, and were married. Additionally, the probability of signing up rate was higher for the migrants in the northeast region who were healthy, had a two-week prevalence, received education on the prevention and treatment of chronic diseases, had a higher scope of mobility (inter-provincial, intra-provincial, and inter-municipal), belonged to an agricultural household, and were married. Further details are provided in Table 4.

**Table 3:** The results of a binary logistic regression analysis for establishing health records of the migrant population in different regions

Features	Variables	Eastern Region		Central Region		Western Region		Northeast Region	
		**OR(95%CI) value**	**Significance**	**OR(95%CI) value**	**Significance**	**OR(95%CI) value**	**Significance**	**OR (95% CI) value**	**Significance**
**Tendency characteristics**	Age								
	25–34	0.408 (0.386 ~ 0.432)	< 0.001	0.998 (0.886 ~ 1.124)	0.976	0.636 (0.594 ~ 0.682)	< 0.001	0.463 (0.392 ~ 0.547)	< 0.001
	35–44	0.502(0.473 ~ 0.532)	< 0.001	0.981 (0.861 ~ 1.117)	0.771	0.655(0.609 ~ 0.705)	< 0.001	0.496 (0.418 ~ 0.589)	< 0.001
	45–54	0.407(0.38 ~ 0.435)	< 0.001	0.979 (0.851 ~ 1.216)	0.765	0.611 (0.564 ~ 0.662)	< 0.001	0.429 (0.358 ~ 0.514)	< 0.001
	55–59	0.331 (0.288 ~ 0.38)	< 0.001	1.026 (0.825 ~ 1.276)	0.82	0.612(0.535 ~ 0.7)	< 0.001	0.623 (0.489 ~ 0.793)	< 0.001
	≥ 60	0.388 (0.342 ~ 0.439)	< 0.001	0.951(0.768 ~ 1.178)	0.645	0.769 (0.683 ~ 0.865)	< 0.001	0.689 (0.557 ~ 0.853)	0.001
	Gender	0.767 (0.737 ~ 0.798)	< 0.001	1.178 (1.11 ~ 1.25)	< 0.001	0.913 (0.872 ~ 0.955)	< 0.001	0.88 (0.798 ~ 0.971)	0.011
	Education level								
	Junior High School	0.59 (0.56 ~ 0.622)	< 0.001	1.066(0.97 ~ 1.172)	0.186	0.744 (0.703 ~ 0.788)	< 0.001	0.716 (0.634 ~ 0.81)	< 0.001
	High school/junior high school	0.767 (0.725 ~ 0.812)	< 0.001	1.132(1.019 ~ 1.258)	0.021	0.856 (0.802 ~ 0.914)	< 0.001	0.764 (0.657 ~ 0.887)	< 0.001
	University Specialists	0.738 (0.688 ~ 0.791)	< 0.001	1.137(1.001 ~ 1.292)	0.048	0.903(0.83 ~ 0.982)	0.017	0.693 (0.566 ~ 0.848)	< 0.001
	Bachelor's degree and above	0.715 (0.661 ~ 0.773)	< 0.001	1.289 (1.106 ~ 1.503)	0.001	1.001 (0.902 to 1.111)	0.983	0.807 (0.642 ~ 1.015)	0.067
	Nature of household registration	1.171(1.119 ~ 1.225)	< 0.001	1.084 (1.014 ~ 1.159)	0.019	1.22 (1.151 ~ 1.294)	< 0.001	1.138 (1.012 ~ 1.279)	0.031
	Marital Status	2.793 (2.622 ~ 2.976)	< 0.001	1.158 (1.045 ~ 1.283)	0.005	1.728 (1.618 ~ 1.845)	< 0.001	1.827 (1.584 to 2.107)	< 0.001
	Mobility hours								
	5 ~ 9	0.712 (0.679 ~ 0.746)	0.712	1.023 (0.958 ~ 1.093)	0.492	0.856 (0.813 ~ 0.901)	< 0.001	0.798 (0.708 ~ 0.9)	< 0.001
	≥ 10	0.614 (0.584 ~ 0.645)	0.614	0.959 (0.887 ~ 1.036)	0.288	0.808 (0.764 ~ 0.855)	< 0.001	0.849 (0.756 ~ 0.954)	0.006
	Employment status								
	Employees	0.944(0.881 ~ 1.012)	0.106	0.817 (0.733 ~ 0.912)	< 0.001	0.905 (0.848 ~ 0.967)	0.003	0.959 (0.837 to 1.099)	0.548
	Employers	0.897 (0.804 ~ 1.001)	0.052	0.751(0.64 ~ 0.88)	< 0.001	0.94 (0.838 ~ 1.054)	0.29	1.456 (1.157 to 1.833)	0.001
	Self-employed workers	1.176(1.085 ~ 1.275)	< 0.001	0.901 (0.795 ~ 1.021)	0.102	1.046 (0.934 to 1.166)	0.233	1.055 (0.893 to 1.246)	0.531
	Other	1.263(0.991 ~ 1.61)	0.059	1.148 (0.691 ~ 1.907)	0.595	1.084 (0.895 to 1.312)	0.409	1.177 (0.65 to 2.132)	0.59
	Mobility range								
	cross-provincial	0.53(0.499 ~ 0.564)	< 0.001	1.175 (1.088 ~ 1.27)	< 0.001	1.011 (0.954 ~ 1.07)	0.718	0.971 (0.858 ~ 1.1)	0.647
	Intra-provincial inter-city	0.76(0.708 ~ 0.816)	< 0.001	1.042 (0.977 ~ 1.11)	0.209	0.745 (0.702 ~ 0.791)	< 0.001	1.165 (1.029 ~ 1.319)	0.016
	Reasons for Mobility								
	Business/Work	0.838(0.761 ~ 0.922)	< 0.001	0.731 (0.647 ~ 0.827)	< 0.001	0.738 (0.681 ~ 0.8)	< 0.001	0.786 (0.673 ~ 0.918)	0.002
	Doing Business	0.713(0.637 ~ 0.798)	< 0.001	0.841 (0.728 ~ 0.973)	0.02	0.606 (0.55 ~ 0.669)	< 0.001	0.671 (0.54 ~ 0.833)	< 0.001
	Relocation of family members	0.925 (0.828 ~ 1.033)	0.168	0.807 (0.7 to 0.929)	0.003	0.809 (0.736 ~ 0.889)	< 0.001	0.776 (0.65 ~ 0.926)	0.005
**Enabling Resources**	urban and rural	0.712 (0.662 ~ 0.766)	< 0.001	0.648 (0.566 ~ 0.741)	< 0.001	0.834 (0.765 ~ 0.908)	< 0.001	0.489 (0.389 ~ 0.615)	< 0.001
	New Agricultural Cooperative	0.966 (0.914 ~ 1.02)	0.214	0.996 (0.886 ~ 1.119)	0.945	0.983 (0.914 ~ 1.058)	0.653	0.909 (0.777 ~ 1.063)	0.233
	Urban residents	0.691(0.643 ~ 0.743)	< 0.001	0.697 (0.615 ~ 0.791)	< 0.001	0.751 (0.689 ~ 0.819)	< 0.001	0.629 (0.536 ~ 0.739)	< 0.001
	Urban workers	0.753 (0.716 ~ 0.791)	< 0.001	0.945(0.84 ~ 1.064)	0.349	0.711 (0.657 ~ 0.771)	< 0.001	0.765 (0.655 ~ 0.893)	0.001
	Publicly funded medical care	0.935 (0.836 to 1.045)	0.236	0.823 (0.659 ~ 1.028)	0.086	1.155 (1.005 to 1.327)	0.043	0.782 (0.531 to 1.152)	0.213
	Place of enrollment								
	Local	0.883 (0.78 ~ 0.999)	0.049	1.887 (1.524 ~ 2.335)	< 0.001	1.327 (1.184 ~ 1.486)	< 0.001	–	
	Domicile	0.728 (0.641 ~ 0.826)	< 0.001	1.517 (1.223 ~ 1.882)	< 0.001	1.044 (0.934 to 1.166)	0.449	0.797 (0.704 ~ 0.901)	< 0.001
	Other places	0.735(0.602 ~ 0.897)	0.002	1.478 (1.079 ~ 2.025)	0.015	0.999 (0.81 to 1.232)	0.992	0.605 (0.338 to 1.084)	0.091
	Income level								
	II	0.95 (0.891 ~ 1.013)	0.115	0.844 (0.774 ~ 0.921)	< 0.001	0.939 (0.886 ~ 0.996)	0.035	0.91 (0.805 ~ 1.028)	0.129
	III	0.98 (0.922 ~ 1.041)	0.505	0.896 (0.824 ~ 0.975)	0.011	0.95 (0.895 ~ 1.009)	0.094	0.914 (0.805 ~ 1.039)	0.17
	IV	0.947 (0.896 ~ 1.001)	0.052	0.791(0.731 ~ 0.856)	< 0.001	0.99 (0.933 ~ 1.05)	0.735	0.812 (0.709 ~ 0.929)	0.002
	V	0.864 (0.813 ~ 0.919)	< 0.001	0.72(0.648 ~ 0.799)	< 0.001	0.965 (0.884 ~ 1.054)	0.431	0.765 (0.608 ~ 0.962)	0.022
**Demand factors**	Health education on chronic disease prevention and treatment	1.848 (1.775 ~ 1.923)	< 0.001	2.524 (2.382 ~ 2.673)	< 0.001	1.872 (1.795 ~ 1.953)	< 0.001	2.483 (2.256 to 2.733)	< 0.001
	Self-assessment of health status	1.206 (1 ~ 1.453)	0.05	1.341(1.072 ~ 1.677)	0.01	1.084 (0.956 to 1.228)	0.208	1.164 (0.953 to 1.423)	0.137
	Two-week prevalence	0.983 (0.896 ~ 1.08)	0.725	0.838 (0.713 ~ 0.986)	0.033	1.04 (0.945 ~ 1.143)	0.423	1.092 (0.892 ~ 1.337)	0.391
	Constants	7.957	< 0.001	1.887	0.191	1.98	0.021	18.352	< 0.001

**Table 4:** The results of a binary logistic regression analysis of family doctor contracting services for the migrant population in different regions

**Features**	Variables	Eastern Region		Central Region		Western Region		Northeast Region	
		OR(95%CI) value	Significance	OR(95%CI) value	Significance	OR (95% CI) value	Significance	OR (95% CI) value	Significance
**Tendency characteristics**	Age								
	25–34	0.26 (0.242 ~ 0.28)	< 0.001	1.074 (0.922 ~ 1.252)	0.359	0.428 (0.395 ~ 0.464)	< 0.001	0.301 (0.248 ~ 0.366)	< 0.001
	35–44	0.428(0.4 ~ 0.458)	< 0.001	1.091 (0.924 ~ 1.288)	0.304	0.491(0.452 ~ 0.533)	< 0.001	0.354 (0.292 ~ 0.429)	< 0.001
	45–54	0.369(0.34 ~ 0.4)	< 0.001	1.162 (0.973 to 1.388)	0.097	0.46 (0.42 ~ 0.504)	< 0.001	0.288 (0.234 to 0.354)	< 0.001
	55–59	0.35(0.296 ~ 0.415)	< 0.001	1.344 (1.035 ~ 1.745)	0.027	0.474 (0.404 ~ 0.556)	< 0.001	0.499 (0.381 ~ 0.653)	< 0.001
	≥ 60	0.405 (0.347 ~ 0.473)	< 0.001	1.131 (0.875 ~ 1.463)	0.347	0.586 (0.513 ~ 0.67)	< 0.001	0.423 (0.333 ~ 0.539)	< 0.001
	Gender	0.543 (0.512 ~ 0.575)	< 0.001	1.063 (0.987 to 1.145)	0.108	0.74 (0.699 ~ 0.784)	< 0.001	0.691 (0.613 ~ 0.779)	< 0.001
	Education level								
	Junior High School	0.346 (0.323 to 0.371)	< 0.001	1.04 (0.925 ~ 1.168)	0.513	0.545(0.509 ~ 0.584)	< 0.001	0.539 (0.469 ~ 0.621)	< 0.001
	High School/Junior College	0.626 (0.586 ~ 0.669)	< 0.001	1.156 (1.015 to 1.317)	0.029	0.675(0.625 ~ 0.728)	< 0.001	0.596 (0.502 ~ 0.707)	< 0.001
	University Specialists	0.636 (0.584 ~ 0.692)	< 0.001	1.329 (1.137 ~ 1.554)	< 0.001	0.753(0.683 ~ 0.831)	< 0.001	0.414 (0.323 ~ 0.53)	< 0.001
	Bachelor's degree and above	0.67(0.608 ~ 0.738)	< 0.001	1.455 (1.207 to 1.754)	< 0.001	0.84(0.745 ~ 0.948)	0.005	0.573 (0.436 ~ 0.752)	< 0.001
	Nature of household registration	1.713 (1.603 ~ 1.832)	< 0.001	1.3 (1.194 ~ 1.416)	< 0.001	1.073 (0.999 ~ 1.153)	0.054	1.177 (1.02 to 1.357)	0.026
	Marital Status	4.886 (4.427 ~ 5.392)	< 0.001	1.27(1.112 ~ 1.449)	< 0.001	2.389 (2.192 ~ 2.605)	< 0.001	2.586 (2.144 to 3.121)	< 0.001
	Mobility hours								
	5 ~ 9	0.511 (0.478 ~ 0.547)	< 0.001	1.095 (1.009 to 1.189)	0.03	0.7 (0.657 ~ 0.746)	< 0.001	0.613 (0.53 ~ 0.708)	< 0.001
	≥ 10	0.333 (0.309 ~ 0.359)	< 0.001	1.112 (1.01 to 1.223)	0.03	0.565 (0.526 ~ 0.628)	< 0.001	0.586 (0.51 to 0.674)	< 0.001
	Employment status								
	Employees	0.969 (0.885 ~ 1.062)	0.507	0.661(0.582 ~ 0.751)	< 0.001	0.908 (0.839 ~ 0.982)	0.016	0.835 (0.711 ~ 0.981)	0.028
	Employers	0.868 (0.739 ~ 1.02)	0.085	0.7(0.577 ~ 0.848)	< 0.001	0.935 (0.805 ~ 1.086)	0.38	1.467 (1.116 ~ 1.927)	0.006
	Self-employed workers	1.159 (1.044 ~ 1.286)	0.006	0.756 (0.652 ~ 0.876)	< 0.001	1.196 (1.095 ~ 1.306)	< 0.001	1.115 (0.918 ~ 1.355)	0.273
	Other	0.872 (0.624 ~ 1.22)	0.425	0.722 (0.382 ~ 1.365)	0.316	1.015 (0.81 to 1.271)	0.9	0.947 (0.476 ~ 1.882)	0.876
	Mobility range								
	cross-provincial	0.54(0.501 ~ 0.582)	< 0.001	1.231(1.121 ~ 1.352)	< 0.001	1.004 (0.938 ~ 1.074)	0.916	1.141(0.984 ~ 1.323)	0.081
	Intra-provincial inter-city	0.696 (0.634 ~ 0.763)	< 0.001	1.019(0.942 ~ 1.103)	0.637	0.69 (0.641 ~ 0.742)	< 0.001	1.164 (1 ~ 1.355)	0.05
	Reasons for Mobility								
	Business/Work	0.768(0.679 ~ 0.869)	< 0.001	0.536(0.467 ~ 0.614)	< 0.001	0.709 (0.646 ~ 0.779)	< 0.001	0.683 (0.571 ~ 0.816)	< 0.001
	Doing Business	0.515(0.443 ~ 0.599)	< 0.001	0.468 (0.397 ~ 0.552)	< 0.001	0.433 (0.385 to 0.487)	< 0.001	0.374 (0.287 to 0.487)	< 0.001
	Relocation of family members	0.844(0.733 ~ 0.973)	0.019	0.639(0.546 ~ 0.747)	< 0.001	0.881 (0.79 ~ 0.982)	0.023	0.613 (0.498 ~ 0.754)	< 0.001
**Enabling Resources**	urban and rural	0.821(0.748 ~ 0.901)	< 0.001	0.646 (0.545 ~ 0.765)	< 0.001	0.85 (0.767 ~ 0.942)	0.002	0.675 (0.518 ~ 0.878)	0.003
	New Agricultural Cooperative	1.122 (1.047 ~ 1.203)	0.001	0.905 (0.776 ~ 1.055)	0.202	0.98(0.897 ~ 1.071)	0.654	0.939 (0.783 ~ 1.127)	0.501
	Urban residents	0.748(0.681 ~ 0.82)	< 0.001	0.826 (0.702 ~ 0.971)	0.02	0.883(0.795 ~ 0.98)	0.019	0.69 (0.574 ~ 0.83)	< 0.001
	Urban workers	0.95(0.891 ~ 1.013)	0.12	1.109 (0.952 to 1.292)	0.182	0.868 (0.788 ~ 0.957)	0.004	0.979 (0.815 ~ 1.176)	0.819
	Publicly funded medical care	0.924(0.8 ~ 1.066)	0.278	0.734 (0.545 ~ 0.989)	0.042	0.815 (0.686 ~ 0.969)	0.02	1.014(0.653 ~ 1.575)	0.952
	Place of enrollment								
	Local	0.928 (0.792 ~ 1.088)	0.358	3.607 (2.563 ~ 5.076)	< 0.001	1.217 (1.061 ~ 1.394)	0.005		
	Domicile	0.792(0.675 ~ 0.93)	0.005	2.566 (1.817 ~ 3.622)	< 0.001	0.955 (0.836 ~ 1.091)	0.501	0.785(0.678 ~ 0.909)	0.001
	Other places	0.783 (0.608 ~ 1.088)	0.058	2.535 (1.609 ~ 3.995)	< 0.001	0.969 (0.753 to 1.245)	0.804	0.771 (0.385 ~ 1.544)	0.464
	Income level								
	II	0.908(0.838 ~ 0.985)	0.02	0.778 (0.701 ~ 0.864)	< 0.001	0.996 (0.928 ~ 1.069)	0.918	0.897 (0.776 ~ 1.037)	0.143
	III	0.936(0.867 ~ 1.01)	0.088	0.776 (0.701 ~ 0.859)	< 0.001	1.056 (0.983 to 1.135)	0.136	0.967 (0.832 ~ 1.124)	0.659
	IV	0.864 (0.806 ~ 0.927)	< 0.001	0.691 (0.628 ~ 0.761)	< 0.001	1.078 (1.004 to 1.157)	0.039	0.801(0.681 ~ 0.942)	0.007
	V	0.742 (0.685 ~ 0.803)	< 0.001	0.639 (0.56 ~ 0.729)	< 0.001	1.104 (0.994 ~ 1.226)	0.066	0.754 (0.573 ~ 0.993)	0.045
**Demand factors**	Health education on chronic disease prevention and treatment	1.474(1.399 ~ 1.552)	< 0.001	2.59 (2.416 ~ 2.777)	< 0.001	1.784 (1.696 ~ 1.877)	< 0.001	2.483 (2.219 ~ 2.779)	< 0.001
	Self-assessment of health status	1.138(0.893 ~ 1.449)	0.296	1.241 (0.956 to 1.61)	0.104	1.126 (0.97 ~ 1.308)	0.119	1.226 (0.968 ~ 1.554)	0.091
	Two-week prevalence	0.974 (0.862 ~ 1.099)	0.664	0.858 (0.699 ~ 1.053)	0.142	1.025 (0.914 ~ 1.149)	0.675	1.208 (0.958 ~ 1.525)	0.111
	Constants	1.316	0.352	0.414	0.179	1.512	0.255	2.864	0.155

### Results of Shapley value decomposition of basic public health service utilization for migrant population in different regions

The Shapley value decomposition results showed that the dimensions of the Anderson model had different contribution rates for explaining the establishment of health records of migrants in different regions. The eastern region exhibited the highest percentage of contribution from propensity characteristics, reaching up to 71.49%. This finding highlighted the significance of propensity characteristics as the primary factor influencing the establishment of migrant population records in the eastern region. In the western region, propensity characteristics and need factors showed similar explanatory strengths, accounting for 48.59% and 43.5%, respectively, in the establishment of health records for the migrant population. In contrast, the need factor exhibited the highest contribution rate in the central and northeastern regions, accounting for 70.2% and 59.25%, respectively. These findings showed the importance of the need factor in establishing the health records of migrants in these two regions. Among the individual factors affecting the migrant population, the most significant contributor to establishing health records across all regions was health education regarding chronic disease prevention and treatment. The contribution rates varied by region: Eastern (21.28%), Central (69.44%), Western (43.5%), and Northeastern (59.25%). In the Eastern region, marital status (19.59%), age (18.45%), and gender (15.34%) emerged as the three most pivotal factors influencing the disparities in health record establishment among the migrant population. In the Central region, health insurance (11.28%) and participation location (6.93%) were the key factors influencing health record establishment. These factors also played a significant role in explaining differences in health record establishment among migrant populations in the Western and Northeastern regions, as illustrated in Table 5. The results of the Shapley value decomposition of the differences in appointing a family doctor among the migrant population in different regions are presented in Table 6. The dimensions of Anderson's model had slightly different contribution rates in explaining the appointment of family doctors among the migrant population in each region. Propensity characteristics played a key role in the eastern, western, and northeastern regions, with contribution rates of 93.72%, 69.84%, and 57.18%, respectively. The demand factor was the most influential feature affecting the variation in appointing a family doctor among the migrant population in the central region (55.96%). Among individual variables, the length of mobility (34.16%) was the strongest explanatory factor for the appointment of a family doctor among the migrant populations in the eastern, central, western, and northeastern regions. Other significant factors included the place of participation (9.78%), gender (14.15%), and length of mobility (17.68%) for the respective regions.

**Table 5:** Decomposition results of Shapley values established in the health records of migrants in different regions

Feature	variable	East		central		West		Northeast	
		**Shapley**	**Contribution rate(%)**	**Shapley**	**Contribution rate(%)**	**Shapley**	**Contribution rate(%)**	**Shapley**	**Contribution rate(%)**
Propensity	Age	0.0110	18.45			0.0008	2.2	0.0002	0.34
	Gender	0.0092	15.34	0.0012	2.41	0.0013	3.54	0.0017	3.34
	Education	0.0005	0.87			0.0002	0.55	0.0006	1.16
	Nature of household registration	0.0030	5.01	0.0001	0.17	0.0016	4.2	0.0007	1.45
	Marital status	0.0117	19.59	0.0006	1.19	0.0040	10.89	0.0040	7.85
	Range of migration	0.0071	11.94			0.0005	1.44		
	Reason for migration	0.0002	0.29	0.0023	4.56	0.0011	2.88	0.0013	2.54
	Duration of migration					0.0035	9.54	0.0016	3.18
Enabling	medical insurance	0.0040	6.65	0.0057	11.28	0.0049	13.35	0.0075	14.54
	Income level			0.0017	3.26			0.0012	2.4
	Insured places	0.0003	0.56	0.0035	6.93	0.0020	5.4	0.0019	3.64
Need	Health education	0.0127	21.28	0.0350	69.44	0.0160	43.5	0.0304	59.25
	Self-rated health status			0.0002	0.36				
	two-week prevalence			0.0002	0.4				
total		0.0597	100	0.0504	100	0.0369	100	0.0512	100

**Table 6:** Decomposition results of the Shapley value of the appointed family doctors of migrants in different regions

**Feature**	variable	East		Central		West		Northeast	
		Shapley	rate(%)	Shapley	rate(%)	Shapley	rate(%)	Shapley	rate(%)
Propensity	Age	0.0144	9.57			0.0033	5.67	0.0010	1.29
	Gender	0.0304	20.2			0.0082	14.15	0.0094	12.72
	Education	0.0015	0.98	0.0010	1.6	0.0010	1.68	0.0050	6.72
	Nature of household registration	0.0164	10.85	0.0014	2.36			0.0031	4.17
	Marital status	0.0227	15.08	0.0012	1.96	0.0079	13.78	0.0092	12.44
	Range of migration	0.0039	2.6	0.0003	0.47	0.0013	2.2		
	Duration of migration	0.0515	34.16	0.0009	1.43	0.0173	30	0.0131	17.68
	Reason for migration	0.0004	0.28	0.0055	9	0.0007	1.2	0.0016	2.16
	Employment status			0.0033	5.44	0.0007	1.16		
Enabling	Insured places	0.0016	1.03	0.0035	5.68	0.0027	4.64	0.0038	5.1
	Income level	0.0015	0.96	0.0038	6.15			0.0011	1.45
	Insured places			0.0060	9.78			0.0016	2.17
Need	Health education	0.0053	3.51	0.0343	55.96	0.0124	21.46	0.0253	34.11
total		0.1507	100	0.0612	100	0.0576	100	0.0742	100

## Discussion

In this study, we assessed the utilization of basic public health services by the migrant population in China, using a nationally representative sample. We focused on the establishment of health records and family doctor contracting services for the migrant population in different regions of China. We also investigated the barriers and factors that affect the low utilization rate of these services, as well as, the weights and differences of each influencing factor in the two services. The results of the analysis revealed the differences and similarities in the mechanisms of influence on the migrant population, specifically the contribution of each influencing factor. Given that China is a developing country with the largest migrant population in the world, this empirical evidence is of significant practical importance.

The results of this study showed that the migrant population had different levels of utilization of different types of basic public health services. This finding was similar to those of previous studies [21]. The establishment rate of health records for the migrant population in China was 33.18%, which was an increase of 14.02% compared to similar findings in another study (2017) [41], where it was 29.1%. However, it was significantly lower than the establishment rate of 80.6% reported in another study [42] for urban and rural residents in China in 2016. The signing rate of family doctors for the migrants in China was even lower, with a rate of 18.70%. This fell short of the target of 30% proposed in the Guidance Opinions on Promoting Family Physician Signing Service [23]. There was a significant disparity between regions. The lower rates of establishment of medical files and appointment of doctors among the migrant population flowing into the eastern region might be attributed to two factors. First, the migrant population in this region was more dispersed and larger in number, which made it challenging to establish health files and contract family doctors. Second, the eastern region had better healthcare facilities and provided the migrant population with more options for medical care. This difference further complicated the process of the establishment of health files and appointment of family doctors for the migrants. We found that many individuals were unaware of or uninterested in maintaining a health record. This suggested that this particular group of migrants lacked knowledge or concern regarding health record construction and highlighted the need for improvement in this area. These findings were similar to those of other studies conducted in China [43].

The influencing factors of different types of basic public health services were common and different. Propensity characteristics such as age, gender, and education level were the most common influencing factors [43], while the enabling resources and demand factors played a smaller role. The establishment of health records and the appointment of family doctors varied significantly based on the sociological characteristics of the migrant population and between regions, which were similar to the findings of previous studies [26]. We found that women, individuals with at least a junior high school education, and those with higher income levels in the eastern region were more inclined to create health records, which was also reported in another study [27]. However, the willingness to create health records based on marriage and enrollment in a health insurance plan was not observed in the eastern region but was observed in other regions. The higher rate of health record creation among married individuals was similar to that reported in another study [24]. This could be attributed to the stronger sense of family responsibility among married individuals, which led to a greater influence of family on health-related behaviors. We also found that health education on the prevention and treatment of chronic diseases positively contributed to the construction of health records in the migrant population, which matched the results of another study [41]. Our results indicated that the nature of hukou affected the utilization of basic public health services for the migrant population, particularly in the central and western regions of the country. This finding was similar to that reported in another study [26]. Additionally, participation in health insurance was found to play a significant and positive role in establishing health records for migrants. The probability of establishing a health record was higher for migrants enrolled in an area of influx compared to those enrolled elsewhere; these findings were similar to those reported in other studies [44, 45]. However, contrary to another study [46] that found no effect of health insurance type on health service utilization among the migrant population, we found that the same type of health insurance in different regions significantly affected the health service utilization of the migrant population. We found that the signing rate of health records was higher among the migrant population with good self-rated health compared to those with poor self-rated health. This suggested that the unhealthy migrant population probably had difficulty understanding the content of basic public health services and were less likely to avail of such services. This finding was similar to that reported in another study [47]. We also found that the distance of mobility influenced the rate of contracting family doctor services. Specifically, the signing rate was lower in regions with longer migration distances, such as the western and northeastern regions. This could be attributed to the greater difficulty in providing family doctor contracting services due to the distance of relocation, which in turn affected the willingness of the migrant population to sign up and the continuation of the services provided. Health education positively affected the engagement of the migrant population with family doctors. However, the utilization rate of contracted family doctor services was found to be lower among migrants with lower incomes. This finding was similar to those of other studies [48, 49]. The lower utilization rate might be attributed to the fact that these groups may not prioritize their health and may not be aware of the availability of family doctor contracting services in the regions they migrated to. Thus, they may lack the motivation to participate in such services, resulting in a lower level of utilization. Most factors responsible for the large differences between the two basic public health services varied considerably in the central region, which might be related to the high rate of health records of residents in the region and the fastest growth rate of total services in the central region [50].

The influencing mechanisms of propensity characteristics, enabling resources, and demand factors in the two types of essential public health services showed some differences. Additionally, the utilization of basic public health services by the migrant population in different areas was affected by a combination of propensity characteristics, enabling resources, and demand factors. Tendency characteristics and demand factors were the two main dimensions that influenced this. Among the demand factors, the level of health education for chronic disease prevention and treatment played a key role in directly affecting the establishment of health records and the appointment of family doctors by the migrant population. Additionally, enabling resources in medical insurance was the major variable that affected the establishment of health records for the migrant population. This is because health education, which is an important component of basic public health services, promotes the construction of health records for the migrant population [41]. Migrant populations with medical insurance might be more likely to seek information on their health status and have a greater need for health information, which was also reported in other studies [51]. Among the factors influencing family doctor contracting services among the migrant population, the propensity characteristic dimension had the strongest explanatory power. The variables that had a strong influence were health education on chronic disease prevention and treatment, length of mobility, and gender. Health education and length of mobility positively contributed to family doctor contracting services among migrants. A study [52] found that women have higher health service needs than men, and our results showed that women were more likely than men to sign up for family doctor services in all regions. The willingness of migrants of different ages, marital status, education levels, and mobility characteristics to enroll in family doctor services need to be considered.

A study [26] found that basic public health services provided free of charge were more appealing to the migrants in the central and western parts of China, compared to the more economically developed eastern part. These individuals were more interested in learning about relevant policies, cooperating with relevant departments to establish health records, appointing a family doctor, and receiving health education on chronic diseases. A study [53] also found that the acceptance rates of health education among the migrant population may not correlate positively with regional economic differences, which matched our findings. The acceptance rate of health education for the prevention and treatment of chronic diseases among migrants was higher in the central and western regions compared to that in the eastern and northeastern regions. This difference might be related to the allocation of public health expenses in China, which is generally based on the headcount of the regional household population. The eastern region, which has a larger migrant population, does not receive additional funds, which leads to poorer per capita health education [54]. Therefore, it is important to prioritize addressing the shortcomings in the northeastern region in terms of insurance participation [29] and funding for health education. This can be achieved by appropriately increasing financial subsidies in the eastern region to decrease the geographical gap. The above-mentioned strategy might improve the effectiveness of health services for the migrant population and ensure equal access to health services for the migrant population across all regions.

The results obtained in this study and related studies indicated that regional factors might affect the health services available to the migrant population. Our findings suggested that the management of health record information and family doctor contracting services need to be improved specifically for the migrant population. This improvement should focus on key dimensions and influencing factors and include targeted efforts to strengthen publicity and education. It is important to obtain more information on the migrant population regarding the equalization of services by considering their education level, demographic characteristics, and mobility patterns. A study [55] highlighted that the basic public health service financing policy implemented in China, which is based on the resident population, may inadvertently and legally allow regions to avoid taking the responsibility of providing basic public health services to migrants. This issue can hinder the equalization of service provision for the migrant population. To meet the health service needs of the migrant population, appropriate health measures need to be implemented based on the distribution of public health resources in different areas.

## Limitations

This study had some limitations. Due to the use of self-reported information from survey respondents, there might have been recall bias in the utilization of basic public health services, resulting in an underestimation of the utilization of public health services. Another limitation includes the difficulty in accounting for all relevant influencing factors. Additionally, other confounding factors that obscure the true relationship between these factors and the utilization of public health services need to be further investigated.

## Conclusions

The migrant population has different levels of utilization of various basic public health services. However, there is a big gap between different regions. The utilization of basic public health services by migrants is affected by multiple factors, and the level of utilization of basic public health services by migrants with different characteristics is different and unbalanced in different regions. Second, we found that migrants were unwilling to participate in insurance schemes, invest in health education, and utilize inter-group health services. The Shapley value decomposition method showed differences in the influence mechanism of propensity characteristics, which enabled resources and demand factors in the two service types and identified propensity characteristics and demand factors as the two key dimensions with the highest explanatory power. The most influential factor was health education for chronic disease prevention and treatment. Therefore, it is necessary to break down regional barriers, and the state needs to differentiate the allocation of public resources in various regions to alleviate the pressure on migrant population gathering areas. Policy priorities and resource inputs can be considered in different regions. The migrant population with different characteristics has different needs for basic public health services, and the corresponding organizational resources should be constantly changed. Concerning the existing policies of China, the central and provincial finance grants special funds for basic public health services to the eastern, central, and western regions in different proportions. Second, starting from the actual needs of the migrant population, different resource combinations were formed, focusing on male migrants, low education level, unmarried status, not interested in medical insurance, agricultural household registration, large mobile range, short mobile time, not receiving health education, and other mobile groups, to establish targeted basic public health service projects. Additionally, it can strengthen the pertinence and effectiveness of publicity and education, combine the characteristics of the migrant population, and cooperate with relevant health policies, such as the construction of urban medical union, the settlement strategy of long-distance medical treatment, online medical services, etc., to more effectively guide the migrant population to take the initiative to establish resident health files, appoint family doctors, receive education on the prevention and treatment of chronic diseases, and other basic public health services. Thus, it can help achieve the equality of basic health services for the migrant population.

## Data Availability

Available datasets were analyzed in this study. The data used in this study are from the Migrant Population Service Center, National Health Commission of China (NHCC).
